# Ordering of Polystyrene Nanoparticles on Substrates Pre-Coated with Different Polyelectrolyte Architectures

**DOI:** 10.3390/ijms140612893

**Published:** 2013-06-20

**Authors:** Zuleyha Yenice, Matthias Karg, Regine von Klitzing

**Affiliations:** 1 Department of Chemistry, Stranski-Laboratorium, Technische Universität Berlin, Strasse des 17. Juni 124, D-10623 Berlin, Germany; E-Mail: yenice@mailbox.tu-berlin.de; 2 Physical Chemistry I, University of Bayreuth, Universitaetsstr. 30, D-95440 Bayreuth, Germany; E-Mail: matthias.karg@uni-bayreuth.de

**Keywords:** self-assembly, polystyrene nanoparticles, dip-coating, spin-coating, non-close packed, polyelectrolyte brushes, PDMAEMA brushes, multilayer films, particle deposition, interface

## Abstract

Adjusting the inter-particle distances in ordered nanoparticle arrays can create new nano-devices and is of increasing importance to a number of applications such as nanoelectronics and optical devices. The assembly of negatively charged polystyrene (PS) nanoparticles (NPs) on Poly(2-(dimethylamino)ethyl methacrylate) (PDMAEMA) brushes, quaternized PDMAEMA brushes and Si/PEI/(PSS/PAH)_2_, was studied using dip- and spin-coating techniques. By dip-coating, two dimensional (2-D), randomly distributed non-close packed particle arrays were assembled on Si/PEI/(PSS/PAH)_2_ and PDMAEMA brushes. The inter-particle repulsion leads to lateral mobility of the particles on these surfaces. The 200 nm diameter PS NPs tended to an inter-particle distance of 350 to 400 nm (center to center). On quaternized PDMAEMA brushes, the strong attractive interaction between the NPs and the brush dominated, leading to clustering of the particles on the brush surface. Particle deposition using spin-coating at low spin rates resulted in hexagonal close-packed multilayer structures on Si/PEI/(PSS/PAH)_2_. Close-packed assemblies with more pronounced defects are also observed on PDMAEMA brushes and QPDMAEMA brushes. In contrast, randomly distributed monolayer NP arrays were achieved at higher spin rates on all polyelectrolyte architectures. The area fraction of the particles decreased with increasing spin rate.

## 1. Introduction

Assembly of colloidal building blocks is a promising bottom-up concept for the fabrication of new functional materials, which are of interest in a broad range of areas such as nanoelectronics, photovoltaics, spintronics and sensing. For such applications the assembly process must allow for control of the surface coverage, center-to-center particle distance and the degree and type of order.

The 3-D or 2-D particle assemblies can be either densely packed (close packed) or non-close packed [1,2]; the inter-particle distance, as well as the symmetry of the ordering, determines the properties of the material.

There are several different techniques to create non-close packed 2-D periodic arrays such as soft lithography, stretching of a film of deposited particles [3,4], swelling the film [5], plasma-induced size reduction of polymeric nanoparticle monolayers [6], and spin-coating combined with UV-polymerization. The latter technique creates hexagonal non-close packed 2-D and 3-D particle arrays in a polymer matrix, for the fabrication of periodic nanostructured materials [7,8], biomimetic antireflection coatings [9,10] and for other optical applications [11,12] on large scales. All of these methods, to produce non-close packed ordered arrays, require further modification after the self-assembly process. However, there is a lack of fundamental understanding of the self-assembly process, which therefore limits our ability to improve the fabrication techniques.

In this study we use spin- and dip-coating as bottom-up approaches to examine the impact of different substrates on the particle density of the deposited layer.

Bottom-up approaches based on NP self-assembly are important tools to create nano devices and gain better fundamental understanding of the interaction between adsorbates and substrates. By examining the impact of adjusting the coating parameters and using substrates with different properties, we can improve our understanding of the process. There are different methods and phenomena used for bottom-up approaches, such as the evaporation-induced method, the rapid deposition method, chemically assisted self-assembly and spin- and dip-coating methods.

A key parameter in self-assembly processes as well as particle deposition is electrostatic interaction. In the case of colloids and substrates of opposite charge, attractive interactions can lead to well-ordered 2-D and 3-D particle arrays [13]. The dip coating process is a good example where electrostatic interactions can play a role depending on the adsorbate and substrate properties.

Dip coating is a popular self-assembly technique, in which a substrate is immersed into a dipping solution for a specific time and is then withdrawn from the suspension. The conventional dipping method utilizes a self-diffusion process of charged species onto an oppositely charged surface in a dilute dipping solution. The dipping process is accompanied by solvent evaporation. The rate of evaporation depends on process parameters such as withdrawal velocity and drying conditions. As the film thickness declines to less than twice the particle diameter, due to solvent evaporation, the vertical component of the capillary forces drags the particles downwards to the substrate where they self-assemble into ordered structures [14].

Another very popular technique for NP self-assembly is the spin-coating technique. When combined with electrostatic adsorption, this technique can lead to fast fabrication of well-ordered 2-D and 3-D NP assemblies [7,15,16]. The rotation speed during spin-coating process is a very important parameter. It was reported that the surface coverage of microgel particles on solid surfaces depends strongly on the rotation speed [17]. Spin-coating is also used in various industrial applications. For the self-assembly of NPs, spin-coating is often compared with the dip-coating technique [18] and other wet coating techniques [19]. It is also often compared with the evaporative deposition technique to determine the effect of the substrate wettability [20].

Despite the assembly method used for the preparation of colloidal mono- or multilayer, another parameter that is frequently varied is the surface modification of the solid substrate. Here, softness, roughness and charge can be influenced in different ways such as by the layer-by-layer assembly of polyeletrolytes onto a charged surface (e.g. a silicon wafer). Another approach is based on “grafting to” or “grafting from” protocols to attach or grow polymer brushes from a solid support.

Surface-grafted polymer brushes attract a lot of interest as substrates for NP assembly because of their unique responsive properties. They can be grown from different surfaces by the “grafting from” approach, using different polymerization techniques, such as Surface-Initiated Atom Transfer Radical Polymerization (SI-ATRP) [21–24], or they can be attached to different surfaces using the “grafting to” approach [25,26]. The approach used depends on the chain length and grafting density (number of chains per unit area of grafted surface) required. By using the “grafting from” approach, densely packed brushes can be achieved.

Polymer brushes are suitable surfaces for NP stabilization and are capable of responding to changes of temperature, solvent polarity, pH, and other stimuli, generally by reversible swelling and deswelling, which can lead to changes in optical properties of adsorbed NPs and opens a new window for nano-devices such as nanosensors and optical devices [27,28]. These studies are mostly based on Au NPs adsorbed on polymer brushes because of their nano-sensing abilities, which can be altered by changing the inter-particle distance between the NPs and the swelling/deswelling properties of the brushes. Polymer brushes also allow for further modifications, such as quaternization and replacing of counterions, for tuning the wettability [29,30]. They also allow for the possibility to generate surface grafted molecular and macromolecular gradients for controlling the self-assembly of NPs [31,32]. Polymer brushes are also known to have lubrication properties [33,34]. However, there is a lack of research regarding the ordering or self-assembly of PS NPs on polymer brushes.

PEM can also stabilize NP. The layer-by-layer self-assembly technique, used for making PEM, is an efficient method for preparing films of a desired composition and functionality. This technique allows for the adsorption of oppositely charged materials like proteins [35] and NPs [13] onto or in between the polyelectrolyte layers. Entropic, van der Waals, steric, and dipolar forces, as well as electric charges on the sterically charged NPs, determine the stoichiometry of structures. The effect of particle size, morphology [36] and zeta potential of the substrate [37] have been studied for latex NPs assembled on PEM. However, there is a lack of research regarding the ordering of PS NPs on PEM and polymer brushes for the production of non-close packed structures.

In this work we present our studies on PS NP assembly on weak and strong polyelectrolyte brushes and polyelectrolyte multilayers using dip- and spin-coating techniques. This allows us to examine the impact of different substrates, as well as the dip- and spin-coating techniques, on the particle density of the deposited layer. The surface topology of the assembled PS NP arrays was examined by AFM and SEM techniques. Surface coverage and the inter-particle distance of the NP arrays were measured using ImageJ (Image processing program, developed at the National Institutes of Health, Bethesda, MD, USA). Spin-coating with different spin rates (300 rpm, 2000 rpm and 5000 rpm) was used to examine the effect of rotation speed. The surface roughness and the wettability of the surfaces were also considered and measured.

## 2. Results and Discussion

The key objective of the present work was the investigation of the effect of surface modification as well as the deposition technique (spin- and dip-coating) on the assembly of negatively charged PS NP. We were primarily interested in the resulting inter-particle distance (center-to-center) of nearest neighbors, and the degree and type of order. Therefore, negatively charged PS NPs (average diameter by DLS: 208 nm) were deposited on oppositely charged polymer films, *i.e*., polyelectrolyte multilayers and polyelectrolyte brushes, using the dip- and spin-coating techniques, to examine the impact of different substrates and deposition techniques on the particle density of the deposited layer.

### 2.1. Particle Characterization

**Figure 1:**
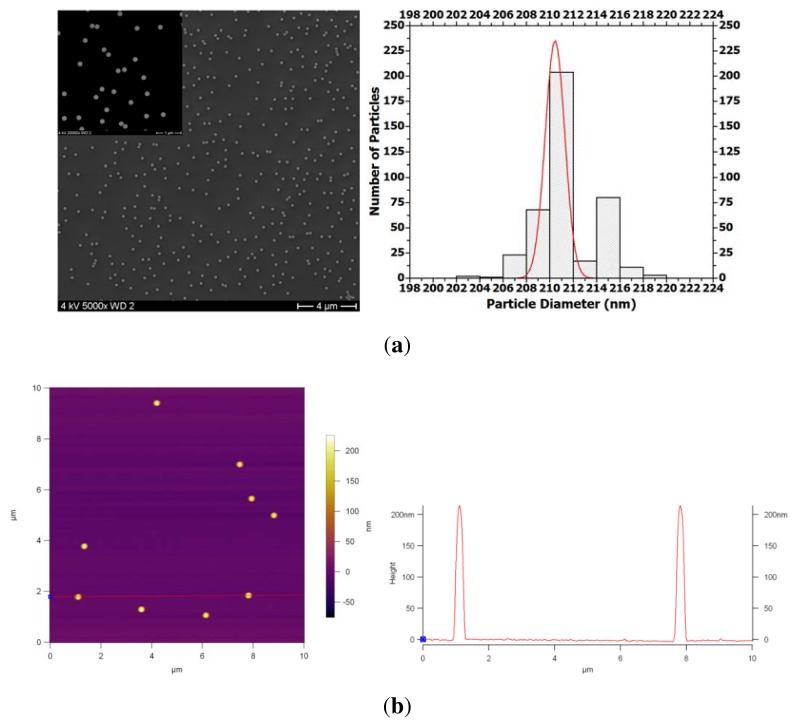
SEM image and the corresponding particle size distribution histogram of PS NPs, fitted with a Gaussian curve (**a**); AFM height profile of the PS NPs together with the cross-sectional diagram with peak heights (**b**).

The average particle size was determined using DLS, SEM and AFM. DLS was used as a scattering technique to study the particle dimensions from ensemble and not from a rather limited number of particles as accessible from imaging techniques. The hydrodynamic radius values were obtained by analyzing the scattering intensity correlation functions with a cumulant fit [38]; we calculated an average hydrodynamic diameter of 208 nm with a polydispersity index of 0.01. For SEM, PS NPs were spin coated onto a bare silicon wafer. The particle size distribution (from SEM results) fitted with a Gaussian curve (solid curve) gave an average size of 210 nm (Figure 1(a)). The particles were measured using the ImageJ software. The cross-sectional diagram with peak heights, collected from the AFM image of the PS NPs, shown in Figure 1(b), confirms the size distribution results.

### 2.2. Characterization of Surface Modifications

**Figure 2:**
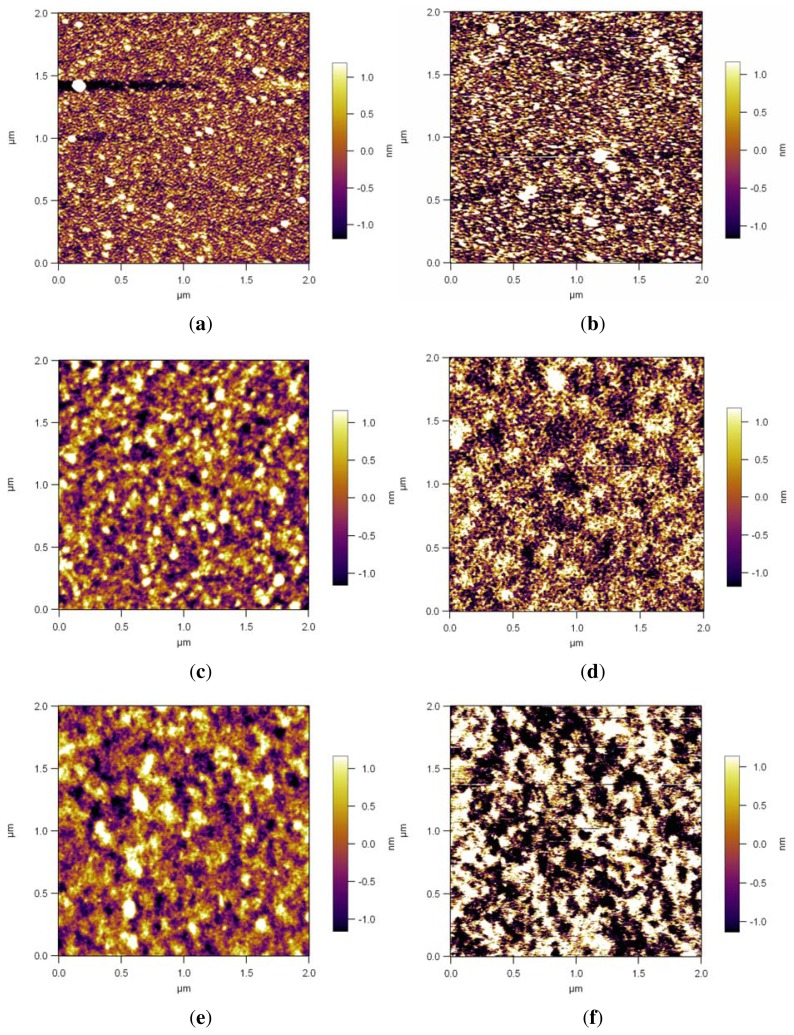
AFM height profiles of Si/PEI/(PSS/PAH)_2_ in ambient conditions, roughness 0.71 nm (**a**) and in water, roughness 1.16 nm (**b**); PDMAEMA brushes in ambient conditions, roughness 0.67 nm (**c**) and in water, roughness 1.70 nm (**d**); QPDMAEMA brushes in ambient conditons, roughness 0.70 nm (**e**) and in water, roughness 2.0 nm (**f**).

All thickness and roughness results of the modified surfaces in ambient conditions as well as in Milli-Q water are shown in Table 1. The differences in roughness of the pre-coated films are not very pronounced. The AFM images of the modified surfaces in ambient conditions and in water are shown in Figure 2.

Si/PEI/(PSS/PAH)_2_ had a thickness of 5 nm (refractive index was fixed to 1.50) measured by ellipsometry in ambient conditions, and the roughness measured by AFM was 0.71 nm. In water the thickness increased to 6.9 nm and the roughness increased to 1.16 nm, as seen in Table 1.

After 4 h polymerization time, PDMAEMA brushes were 17.4 nm thick in ambient conditions (refractive index 1.52, fitted) and 34 nm thick in Milli-Q water (refractive index 1.44, fitted), measured by ellipsometry. The PDMAEMA brush had a RMS roughness of 0.67 nm in ambient conditions and 1.7 nm in water (Table 1).

**Figure 3:**
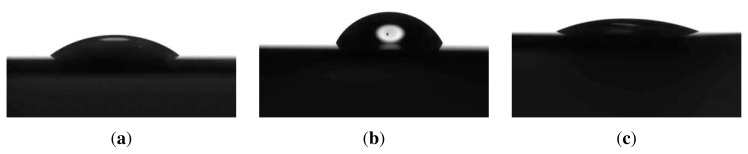
Water CA values of Si/PEI/(PSS/PAH)_2_, 33° (**a**); PDMAEMA brushes, 59° (**b**) and QPDMAEMA brushes, 21° (**c**).

The quaternization resulted in a height increase of the brush of 40%, measured by ellipsometry. It was reported that the quaternization of poly(vinylpridine) by *n*-butyl bromide induced the increase of the thickness of polymeric films grafted onto silicon surfaces. The increase of the thickness was suggested to be caused by the linkage of the *n*-butyl chains to the polymer, which augmented the molecular weight of repeating units, and the incorporation of bromide as a counteranion into the polymeric layer [39]. Therefore, the thickness increase in our system indicates successful quaternization. The other indication of successful quaternization is the decrease in the water CA value as shown in Figure 3. The quaternized brush had an ambient thickness of 25 nm (refractive index 1.54, fitted) and a RMS roughness of 0.70 nm in ambient conditions. In water the thickness increased to 59 nm and the roughness increased to 2 nm, showing almost no change in roughness compared with the PDMAEMA brush as shown in Figure 2(f) and Table 1.

Both Si/PEI/(PSS/PAH)_2_ and polyelectrolyte brushes swell in aqueous conditions and in humid environments, which leads to an increase of the polymer film thickness as well as an increase of the surface roughness. The swelling for the double layers and the PDMAEMA brushes is about 35% and 100% respectively. In general, the swelling of polyelectrolyte multilayer depends on the ionic strength and the number of layers [40]. PDAMEMA has pH dependent properties. As known in the literature, it swells dramatically in low pH environments due to protonation [41] and the swelling is also affected by the grafting density [42]. The PDMAEMA brush turned into a strong polyelectrolyte brush by quaternization and the swelling in water increased. QPDMAEMA brushes have additional repulsion due to increased charges along the chains, which causes stronger swelling compared with the pure PDMAEMA brushes [43]. The swelling of the QPDMAEMA brushes is about 140% in Milli-Q water compared with the dry QPDMAEMA brush and about 300% compared with the dry PDMAEMA brush.

### 2.3. NP Assembly by Dip-Coating

**Figure 4:**
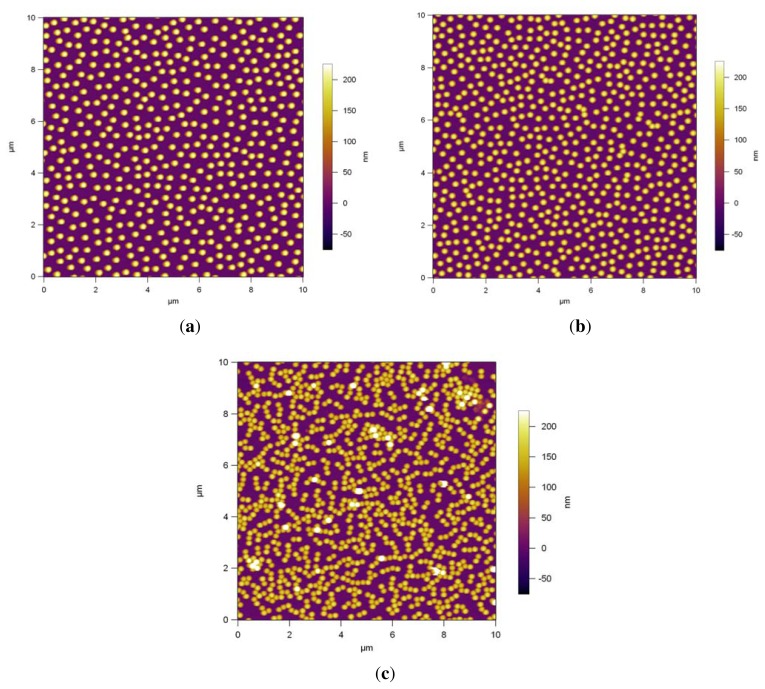
AFM height profiles of assembled PS NPs via dip coating on, Si/PEI/(PSS/PAH)_2_ (**a**); PDMAEMA brushes (**b**); quaternized PDMAEMA brushes (**c**). (**a**) The area fraction is 13.4%; (**b**) The area fraction is 16.3%; (**c**) The area fraction is 24%.

Figure 4 shows AFM images of the dip coated monodisperse PS NPs on the modified surfaces. The NP assembly on Si/PEI/(PSS/PAH)_2_ (Figure 4(a)) results in a randomly dispersed non-close packed monolayer arrangement; the spheres are not in contact. The area fraction is 13.4%. Figure 4(b) shows the AFM image of the assembly of the NPs on PDMAEMA brushes, which also results in a randomly dispersed non-close packed monolayer arrangement where just a few particles are touching each other. The area fraction is 16.3%. Figure 4(c) shows that the deposition onto the QPDMAEMA brushes resulted in randomly distributed particle arrays with aggregation, in which most particles are joined with their nearest neighbor. The surface coverage of the particles is 24%.

**Figure 5:**
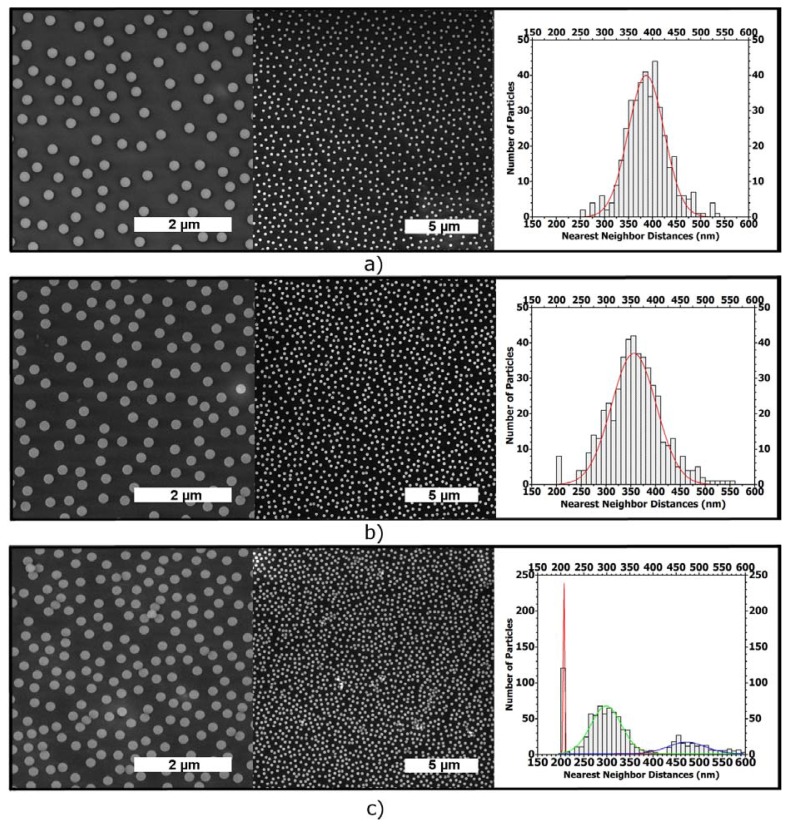
SEM images of assembled PS NPs via dip coating: scale bar 2 *μ*m and 5 *μ*m together with the nearest-neighbor distance graphics fitted with a Gaussian curve, on, Si/PEI/(PSS/PAH)_2_ (**a**); PDMAEMA brushes (**b**); quaternized PDMAEMA brushes (**c**).

In order to study the coverage on larger length scales, we performed SEM measurements of the same samples as used for the AFM investigation. The structures observed by SEM are in very good agreement with the results from AFM. In addition, the low magnification SEM images clearly reveal the homogeneity of the monolayer over large areas. Figure 5 shows the respective SEM images together with the nearest-neighbor distance graphics. The ordering is the same also on 100 *μ*m × 100 *μ*m images. These images are too big to show due to image resolution.

Figure 5(a) shows the assembly of the PS NPs on Si/PEI/(PSS/PAH)_2_. The nearest-neighbor distance, determined between the particle centers, reveals a nearly homogeneous distribution where most of the particles have their nearest-neighbor at a 325 to 450 nm distance. The Gaussian fit curve gives an average inter-particle distance (xc) value of 386.6 nm. The fit curve width at half-maximum height (*ω*) is about 74 nm.

Figure 5(b) shows the SEM images of the assembly of the NPs on PDMAEMA brushes. The inter-particle distance for the vast majority of particles is between 275 nm and 450 nm center to center. The Gaussian fit curve gives an xc value of 356.0 nm and the fit curve width (*ω*) is 90 nm.

Figure 5(c) shows the deposition onto the QPDMAEMA brushes. The nearest-neighbor calculations show clearly the aggregation of the NPs, with many of the particles touching.

PDMAEMA brushes are weak polyelectrolyte brushes having pH dependent properties and are positively charged due to protonation in solutions at pH ≤ 7; the positive charge increases as the pH value declines [43,44]. The brushes were partially charged in the dip-coating solution, which had a pH of 5.55, so the particles could adsorb onto the modified surface, Figure 4(b). PDMAEMA brushes can be converted to strong polyelectrolytes via quaternization using iodomethane as explained above.

It is remarkable that the nearest-neighbor distances, determined for PDMAEMA brushes, are very similar to those determined for the assembled particles onto the Si/PEI/(PSS/PAH)_2_, as shown in Figure 5(a) and (b) . In both cases, most of the particles have their nearest neighbors at the same distances (between 350 and 400 nm). However, the lower *ω* value of the Gaussian fit curve, for the assembled particles on Si/PEI/(PSS/PAH)_2_, indicates a narrower distance distribution and a higher ordering of the particles.

During the dip-coating procedure, the substrate is immersed into the suspension for 4 h. For the self-diffusion process of charged NPs onto oppositely charged surfaces, the electrostatic attraction between the modified wafer surface and the particles and the repulsion between every individual charged particle are important factors. The dipping process is accompanied by solvent evaporation. The rate of evaporation depends on process parameters such as dipping velocity and drying conditions. As the film thickness declines to less than twice the particle diameter, due to solvent evaporation, the vertical component of the capillary forces drags the particles downwards to the substrate where they self-assemble.

A rinsing step in Milli-Q water for 15 min was used after pulling-out the substrate from the suspension. The rinsing step was also used during the layer-by-layer self-assembly of the polyelectrolyte multilayer; the aim of rinsing is the removal of loosely adsorbed polyelectrolytes and particles [45]. The substrate was then dried with a stream of nitrogen. The self-diffusion process of the charged particles on Si/PEI/(PSS/PAH)_2_ and PDMAEMA brushes together with the repulsion between every individual charged particle formed a non-close packed structure as shown in Figure 5(a) and (b).

The repulsion between the charged particles depends on their charge and concentration during their deposition and the properties of the surface on which they are deposited. The quaternized brush has more charged units than the pure PDMAEMA brush, which can lead to a higher electrostatic attraction between the oppositely charged PS NPs and the brush, thereby causing the NPs to aggregate on the brush randomly, as seen in Figure 4(c). The area fraction of 24% is higher than that of the PDMAEMA brushes. We can conclude that high surface charge and more pronounced attraction caused sticking of the particles with no arrangement.

**Figure 6:**
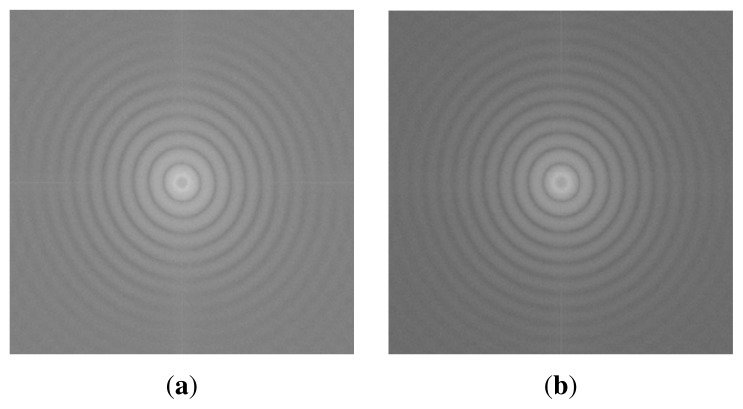
Fourier spectrum of the PS NP self-assembly on Si/PEI/(PSS/PAH)_2_ (**a**) and PDMAEMA brushes (**b**).

The PS NPs were more or less uniformly dispersed without any clustering on the double layers and PDMAEMA brushes, as shown in Figures 4(a) and 4(b) and also in Figure 5. However, as confirmed by the Fourier Transform FFT results in Figure 6, there is no hexagonal symmetry arrangement of the polymeric NPs, because there are no Bragg peaks, but rings, the rings are related to the form factor of the particles. This is possibly due to the non-uniform distribution of polyelectrolyte charged groups producing local areas of surface charge. Particle deposition can be used for detecting electrostatic heterogeneity [37]. If this is the case and there is electrostatic heterogeneity on these PEM and brushes, then particle self-assembly techniques without further modification steps may not be the best option to produce non-close packed patterns with a hexagonal symmetry.

### 2.4. NP Assembly by Spin-Coating

Negatively charged PS NPs were spin coated onto oppositely charged Si/PEI/(PSS/PAH)_2_, PDMAEMA and QPDMAEMA brushes.

**Figure 7:**
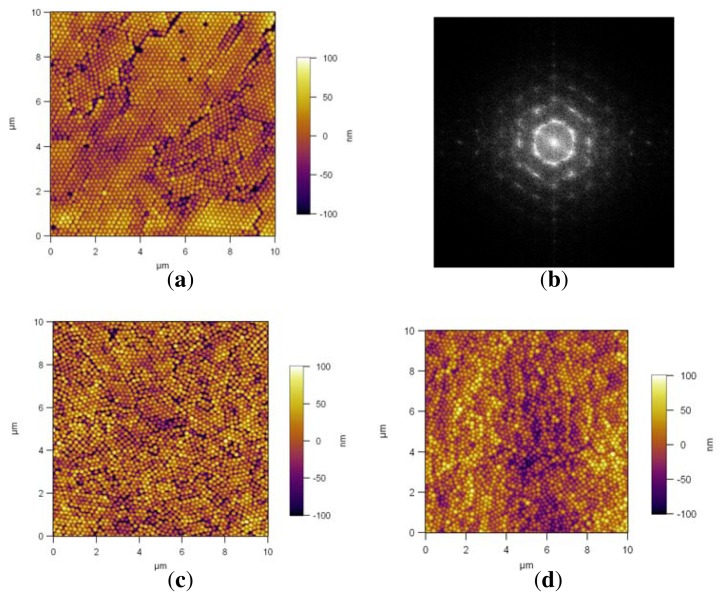
AFM height profiles of PS NP self-assembly by spin coating at 300 rpm, on, Si/PEI/(PSS/PAH)_2_ (**a**), with the corresponding FFT spectrum of the image (**b**); on PDMAEMA brushes (**c**); on quaternized PDMAEMA brushes (**d**).

The AFM images of PS NP assembly prepared at 300 rpm are shown in Figure 7. The slow spin-coating on Si/PEI/(PSS/PAH)_2_ resulted in a close-packed structure, with some defects (Figure 7(a)). The Fourier spectrum from the whole image, shown in Figure 7(b), reveals a hexagonal symmetry of the NP multilayer. Close-packed assemblies with some defects are also observed on PDMAEMA brushes Figure 7(c) and QPDMAEMA brushes Figure 7(d). The relatively slow spin-coating velocity of 300 rpm led to hexagonal close-packed particle arrays with some defects in our study. None of the assembled arrays were defect-free and symmetrically coated for large ranges, and also no assemblies with inter-particle distance could be achieved for this spin rate. To make assembled arrays with an inter-particle distance, 300 rpm was too slow. Higher spin rates were used to see the affect, as seen below.

The RMS roughness of Si/PEI/(PSS/PAH)_2_, PDMAEMA Brushes and QPDMAEMA brushes, measured by AFM, present similar results in ambient conditions (Table 1). In water the roughness increases slightly (Figure 2). Increased roughness can affect the NP movement along the brush while spinning the suspension. The additional charge of the quaternized brush can lead to a higher electrostatic attraction between the brush and particle surface. The question is whether the 200 nm diameter NPs are sensitive to a roughness below 2 nm.

**Figure 8:**
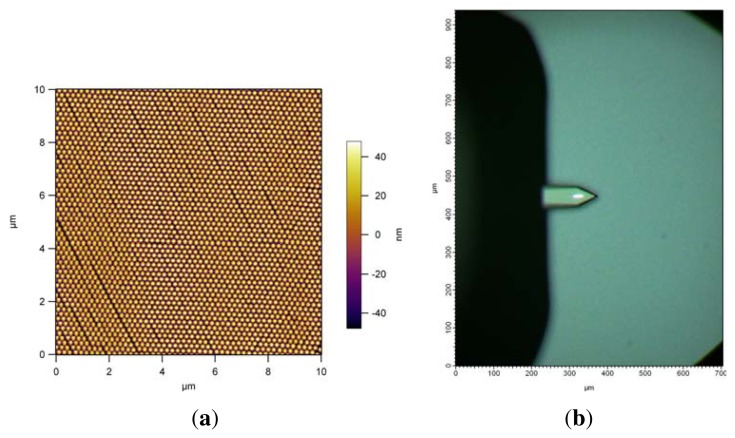
AFM height profile of PS NP monolayer obtained from spin-coating at 300 rpm on a freshly etched Si-wafer (**a**); optical microscopy image of the nanoparticle monolayer prior to investigation by AFM. The uniform coloration due to optical interference from the monolayer manifests the homogeneity of the film (**b**).

The surface hydrophilicity is an important parameter for spin coating, regarding the spreading of the fluid on the surface while spinning. Freshly etched Si-wafers are very hydrophilic; a Milli-Q water drop can totally wet the surface. Slow spin rates on these surfaces can create very symmetrically ordered hexagonal close packed particle arrays with long-range order (see Figure 8(a)). The pattern shown in Figure 8(a) goes over several millimeter ranges, this is confirmed by the homogenous interference color of the pattern shown in Figure 8(b). One of the reasons for the close packed assemblies with defects, fabricated on the modified surfaces, can be the surface hydrophilicity.

In order to determine whether the nanoparticle suspension can wet the wafer surface, we need to examine the surface hydrophilicity. Water CA can be used as a good reference to judge whether we have wetting or not for our suspensions. Water CA measurements produced the following results: 33°, 59°, 21° for Si/PEI/(PSS/PAH)_2_, PDMAEMA brushes and QPDMAEMA brushes respectively. The QPDMAEMA brush has a lower CA value than the pure PDMAEMA brush due to increased charges along the chains. A contact angle less than 90° usually indicates that wetting of the surface is very favorable, but of course freshly etched Si-wafers have perfect wetting properties, which can be the reason for the symmetrical hexagonal close packed arrangement. The CA images of the modified surfaces are shown in Figure 3.

**Figure 9:**
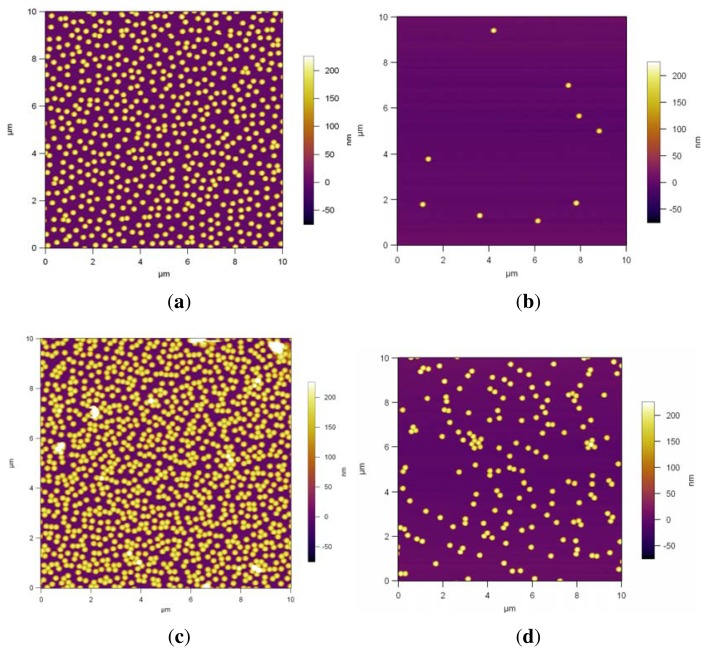

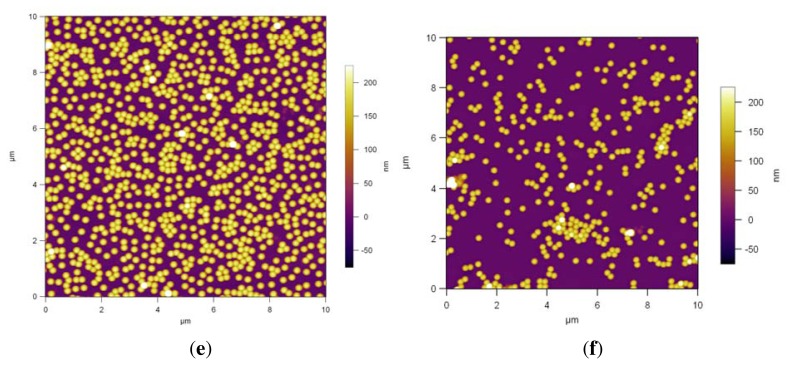
AFM height profiles of PS NP monolayer obtained from spin-coating at 2000 rpm (Left) and 5000 rpm (Right), on, Si/PEI/(PSS/PAH)_2_ (**a**) and (**b**); PDMAEMA brushes (**c**) and (**d**); quaternized PDMAEMA brushes (**e**) and (**f**).

Higher spin rates, 2000 rpm and 5000 rpm, were used to investigate the influence of higher spin rates on the PS NP assembly, and the results are shown in Figure 9. A mixture of 2-D randomly dispersed monolayers and non-close-packed NPs are obtained at 2000 rpm, as seen in Figure 9(a), where the NPs are assembled on Si/PEI/(PSS/PAH)_2_. In the case of PDMAEMA brushes (Figure 9(c)) and QPDMAEMA brushes (Figure 9(e)), monolayers result in randomly adsorbed arrays and random close-packed clusters.

During the spin-coating process the colloidal dispersion spreads out on the substrate and forms a film. This film spreads outward and thins, controlled primarily by centrifugal force and viscous shear force. When the film becomes sufficiently thin, evaporation dominates, further thinning the film. The evaporation step depends on the volatility and other material properties of the coating liquid. For colloidal suspensions, the film thickness during this stage can reduce to the same order as the particle size, at which point capillary forces at the liquid-gas interface can have a significant effect on particle aggregation depending on the spin rate [46,47]. The thickness of the film also depends on the concentration of the NP suspension.

As a general trend from the spin-coating experiments, it can be observed that, independent of the type of substrate, the amount of particles deposited decreases with increasing spin rates. This is expected due to the increasing centrifugal forces and the much faster film thinning for higher spin rates. For 300 rpm, we observe multilayer structures, for 2000 rpm monolayer of low packing densities, and for 5000 rpm the deposition of only very few particles on the substrates. The influence of the polymer architecture on the particle assembly structure is much more pronounced for spin-coating as compared with the previously discussed dip-coating procedure.

The results from increasing the spin rate to 5000 rpm are shown on the right hand side of Figure 9. Figure 9(b) shows that almost all the particles are expelled from the surface of Si/PEI/(PSS/PAH)_2_ due to the high velocity. In the case of PDMAEMA brushes the area fraction of the NPs decreased from 36% (Figure 9(c)) to 5% (Figure 9(d)). For the QPDMAEMA brushes, Figure 9(f), random NP clusters are still visible. As with the double layers and PDMAEMA brushes, the area fraction decreased due to the higher velocity, from 32% to 11%.

In Figure 9, not much difference is seen between the quaternized and pure PDMAEMA brushes at 2000 rpm. This can be attributed to the high spin rates of the spin-coating process; the particles spin too quickly to be affected by the increased surface charge of the QPDMAEMA brush. The greater quantity of NPs on QPDMAEMA brushes at 5000 rpm compared with PDMAEMA brushes can be attributed to the increased swelling of the quaternized brush.

It is known from literature that polymer brushes can have good lubrication properties. It was reported that neutral polymer brushes lead to a massive reduction in sliding friction between the surfaces to which they are attached [33] and that brushes of charged polymers (polyelectrolytes) attached to surfaces rubbing across an aqueous medium, result in superior lubrication, higher than in neutral polymer brushes, where the PE brush-bearing mica surfaces were progressively compressed, and the shear or frictional forces between them were measured [34]. These studies all report a friction decrease and lubricating effect between two polymer brush bearing surfaces. There is a lack of knowledge of the lubricating effect of polymer brushes in the relation of shear-induced ordering of nanoparticles during spin-coating. In our study, we could not observe any increased lubrication effect between the PDMAEMA and QPDMAEMA brushes, towards the PS NP ordering.

## 3. Experimental Section

### 3.1. Synthesis and Characterization of PS NPs

Spherical PS NPs were prepared by surfactant-assisted emulsion polymerization. The polymerization was carried out at 90 °C in an oil bath according to the procedure described in [48] and stored in the fridge at about 4 °C. The particle size and polydispersity was analyzed by Dynamic Light Scattering (DLS) at 25 °C using an ALV/LSE-5004 correlator and an ALV CGS-3 goniometer.

The zeta potential of the PS NPs was determined by using the Zetasizer Nano series Nano-ZS (Malvern Instruments, UK) at room temperature. The suspension was diluted with Milli-Q millipore water. The 0.02% *w*/*v* (mass/volume percentage) suspension used for dip-coating had a pH of 5.55 and the zeta potential of the particles was *−*53.7 mV. The pH of the 0.3% *w*/*v* suspension used for spin coating was 4.8 and the zeta potential of the particles was *−*45 mV.

### 3.2. Surface Modification of Silicon Wafer

The silicon wafers were etched by Piranha solution (H_2_O_2_:H_2_SO_4_; 1:1) for 20 min, then rinsed extensively with Milli-Q water and dried under a nitrogen stream. The silicon oxide layer thickness, measured using ellipsometry in ambient conditions and at different points on the wafer, was 1.3 *±* 0.1 nm. The RMS roughness, measured by AFM using a 1 *μ*m *×* 1 *μ*m box over a 2 *μ*m *×* 2 *μ*m scan area, was about 0.1 nm. These surfaces were then further modified with polyelectrolyte multilayers and brushes for NP deposition purposes.

#### 3.2.1. Polyelectrolyte Multilayers

Branched poly(ethylene imine) (PEI), poly(sodium-4-styrene sulfonate) (PSS) and poly(allyamine hydrochloride) (PAH) were purchased from Aldrich (Steinheim, Germany); the molecular weights were 750,000 g mol*^−^*^1^, 70,000 g mol*^−^*^1^ and 65,000 g mol*^−^*^1^ respectively. NaCl was purchased from Merck. The polyelectrolyte multilayers were deposited onto the silicon wafers by the layer-by-layer self-assembly technique. The wafer was immersed into aqueous solution containing 10*^−^*^2^ M (concentration of monomer units) of the respective polyelectrolytes in Milli-Q water with a salt concentration of 10 *^−^*^1^ M: first in an aqueous solution of PEI (salt free) for 30 min and then subsequently 20 min in each PSS and PAH solutions, letting the multilayers form via this technique. The wafer was rinsed with Milli-Q water three times for 1 min after each deposition step and dried with a stream of nitrogen after the last deposition and cleaning step. Si/PEI/(PSS/PAH)_2_ were prepared as substrates and used for NP deposition.

#### 3.2.2. Weak Polyelectrolyte Brushes (PDMAEMA Brushes)

DMAEMA was purchased from Sigma-Aldrich. The brushes were grown from silicon wafers by the grafting from technique using SI-ATRP. The initiator Amide-BMPUS (2-bromo-2-methyl-*N*-(11-(trichlorosilyl)undecyl)propanamide) was synthesized according to the procedure described in [21] using 10-undecenyl azide instead of *ω*-undecylenyl alcohol. BMPUS with amide groups was used to increase the stability of the polymer brushes [49].

Building of initiator self-assembled monolayer (SAM): A monolayer of the surface attached ATRP initiator (11-(2-bromo-2-methyl propanamide) undecyl trichlorosilane) was deposited onto the silicon surface. Freshly cleaned silicon wafers (with large concentration of surface-bound hydroxyl groups due to the etching of the wafers, required for the attachment of the initiator) were placed into a solution of 10 *μ*L of the initiator in 40 mL of anhydrous toluene (to form a 0.001 wt % initiator solution) and kept in the fridge for 16 h, allowing the initiator molecules to form a monolayer. The wafers were then removed, rinsed with toluene, cleaned by ultrasound in toluene for 1 min, and dried under a nitrogen stream. The SAM, measured using ellipsometry, had a thickness of 2 *±* 0.1 nm in ambient conditions.

General procedure for SI-ATRP of DMAEMA: All chemicals were purchased from Sigma-Aldrich. The initiator deposited silicon wafers were then put into a solution of 80 mL DMAEMA (0.475 mol), 80 mL DMSO, 4.65 g 2.2’-Bipyridine and 1.411 g Cu(I)CL under nitrogen atmosphere. The synthesis was carried out at room temperature for 4 h. The silicon wafers were then removed and rinsed with ethanol, cleaned by ultrasound in ethanol for 3 min and dried under a nitrogen stream.

#### 3.2.3. Quaternization of PDMAEMA Brushes (QPDMAEMA Brushes)

PDMAEMA brushes are weak polyelectrolyte brushes and can be converted to strong polyelectrolytes via quaternization using iodomethane. The quaternization was carried out with 1 M methyl iodide (Sigma-Aldrich) in ethanol at 55 °C for 24 h, according to the procedure described in [42]. Here the tertiary amines turn into quaternary ammonium compounds by alkylation, due to the methyl groups in methyl iodide, where the iodide ions will sit in the brush as counterions.

### 3.3. Deposition of PS NPs on Surface Modified Silicon Wafers

All particle depositions onto our surface-modified substrates were performed in a clean room to avoid contamination of the surfaces. Spin-coating was performed with a P-6708 spin-coater from SCS Spin Coating Systems using aqueous PS NP suspensions of 0.3% *w*/*v*. A drop of the NP suspension was put on the desired substrate and the spin-coater was activated until the surface was found to be dry.

Dip-coating was performed by immersing the modified wafers in the suspension for 4 h. The aqueous NP suspensions of 0.02% *w*/*v* had a pH of 5.5 with a zeta potential value of about *−*50 mV, as mentioned above. The wafers were afterwards taken out and put into Milli-Q water 3 times for 5 min each and dried with a nitrogen stream.

### 3.4. Apparatus and Measurement Procedure

#### 3.4.1. Ellipsometry

All ellipsometric measurements were performed with a polarizer-compensator-sample analyzer (PCSA) ellipsometer, Multiscope from Optrel GbR (Wettstetten, Germany) in NullEllipsometry mode in ambient conditions. The measurements were carried out at an incident angle of 70° (wavelength 632.8 nm). For data handling, the software Elli was used. The data were analyzed by using a 4-layer model, namely air-multilayer or brush–SiO_2_–Si for the modified surfaces.

#### 3.4.2. Atomic Force Microscopy (AFM) Measurements

The instrument used for the AFM measurements was an Asylum Research Cypher Scanning Probe Microscope. The cantilever used for scanning in AC mode in air was AC160TS, produced by Olympus with dimensions of 160 *×* 50 *×* 4.6 *μ*m, made of silicon with a reflective coating of aluminum, a spring constant of 26 N/m and resonance frequency (in air) of 300 *±* 100 kHz. For the measurements in water, Olympus OMCL-TR series cantilevers with a triangular shape, Cr/Au coatings, a resonance frequency of 10 kHz, and a spring constant of 0.02 N/m were used. The scan speed was between 0.5 and 1 Hz/s. Data analysis were performed with the program Igor Pro 6.1.2.1. The roughness was calculated as root mean square, RMS. The images were subjected to processing: leveling via plane correction and optimization of brightness/contrast. The AFM images were also analyzed using ImageJ, for determination of the nearest-neighbor inter-particle distance (center-to-center) from the particle arrays generated using dip-coating.

#### 3.4.3. Contact Angle (CA) Measurements

The instrument used for the CA measurements was an OCA 20 (Data-Physics), equipped with halogen lighting to ensure a homogeneous back lighting, a six-fold power zoom lens and a CCD camera. The CA was determined by the sessile drop method in a saturated atmosphere. The drop volume used for the CA investigations was 10 *μ*L (Milli-Q water).

#### 3.4.4. Scanning Electron Microscopy (SEM) Measurements

SEM was carried out on a Hitachi S-2700 scanning electron microscope at the Zentraleinrichtung Elektronenmikroskopie (ZELMI), TU Berlin.

## 4. Conclusions

The adsorption of PS NPs on substrates, pre-coated with different polyelectrolyte architectures (PEM, PDMAEMA and quaternized PDMAEMA brushes), was studied using dip- and spin-coating techniques.

For spin coating, the most prominent effect was the spin rate (rpm). By increasing the spin rate, the area fraction of the NPs decreased for all pre-coated surfaces due to increased centrifugal forces. The polyelectrolyte architecture affected the degree of the decrease in the area fraction. For the Si/PEI/(PSS/PAH)_2_, the decrease was higher than that for the polyelectrolyte brushes. We assume that the NPs sink partially into the swollen brush and this restricts their mobility on the brushed surfaces. The effect is more prominent for the quaternized brushes when the spin rate is 5000 rpm. The reason for this could be the stronger swelling of the quaternized brush, due to increased charges along the polymer chains. We can conclude that we have a polymer architecture dominating effect by spin-coating. It is assumed that roughness is not an important parameter for the ordering in the present study. The differences in roughness of the pre-coated films are not very pronounced. Furthermore, we could not observe any increased lubrication effect between the PDMAEMA and QPDMAEMA brushes towards the PS NP ordering.

In contrast to spin-coating, the polyelectrolyte architecture was not the dominating affect for the NP deposition by dip-coating. The Si/PEI/(PSS/PAH)_2_ and the PDMAEMA brushes showed similar behavior. It seems that the charge plays the decisive role. The adsorption of the NPs onto Si/PEI/(PSS/PAH)_2_ and PDMAEMA brushes is limited by the inter-particle repulsion and randomly distributed non-close packed arrays could be assembled, whereas the adsorption onto the quaternized PDMAEMA brushes was much stronger due to the increased charges along the brush. One has to take into account the two counteracting forces: the attraction between the particles and the substrate and the repulsion between every individual charged particle. We can conclude that dip-coating is suitable for the fabrication of non-close packed particle arrays.

## Figures and Tables

**Table 1:** Thickness and RMS roughness results of modified surfaces.

Surface Type	Thickness (nm) ambient—in water	Roughness (nm) ambient—in water
Si/PEI/(PSS/PAH)_2_	5.0–6.9	0.71–1.16
PDMAEMA brushes	17.4–34.0	0.67–1.70
QPDMAEMA brushes	25.0–59.0	0.70–2.00
